# Stable and bicistronic expression of two genes in somite- and lateral plate-derived tissues to study chick limb development

**DOI:** 10.1186/s12861-015-0088-3

**Published:** 2015-10-30

**Authors:** Adeline Bourgeois, Joana Esteves de Lima, Benjamin Charvet, Koichi Kawakami, Sigmar Stricker, Delphine Duprez

**Affiliations:** CNRS UMR 7622, IBPS-Developmental Biology Laboratory, F-75005 Paris, France; Sorbonne Universités, UPMC Univ Paris 06, IBPS-Developmental Biology Laboratory, F-75005 Paris, France; Inserm U1156, F-75005 Paris, France; Division of Molecular and Developmental Biology, National Institute of Genetics, and Department of Genetics, SOKENDAI (The Graduate University for Advanced Studies), Mishima, Shizuoka Japan; Institue for Chemistry and Biochemistry, Freie Universitaet Berlin, 14195 Berlin, Germany

**Keywords:** Electroporation, 2A peptide, Tol2, Muscle, Tendon, Limb, Chick

## Abstract

**Background:**

Components of the limb musculoskeletal system have distinct mesoderm origins. Limb skeletal muscles originate from somites, while the skeleton and attachments (tendons and connective tissues) derive from limb lateral plate. Despite distinct mesoderm origins, the development of muscle, skeleton and attachments is highly coordinated both spatially and temporally to ensure complete function of the musculoskeletal system. A system to study molecular interactions between somitic-derived tissues (muscles) and lateral-plate-derived tissues (skeletal components and attachments) during limb development is missing.

**Results:**

We designed a gene delivery system in chick embryos with the ultimate aim to study the interactions between the components of the musculoskeletal system during limb development. We combined the Tol2 genomic integration system with the viral T2A system and developed new vectors that lead to stable and bicistronic expression of two proteins at comparable levels in chick cells. Combined with limb somite and lateral plate electroporation techniques, two fluorescent reporter proteins were co-expressed in stoichiometric proportion in the muscle lineage (somitic-derived) or in skeleton and their attachments (lateral-plate-derived). In addition, we designed three vectors with different promoters to target muscle cells at different steps of the differentiation process.

**Conclusion:**

Limb somite electroporation technique using vectors containing these different promoters allowed us to target all myogenic cells, myoblasts or differentiated muscle cells. These stable and promoter-specific vectors lead to bicistronic expression either in somitic-derived myogenic cells or lateral plate-derived cells, depending on the electroporation sites and open new avenues to study the interactions between myogenic cells and tendon or connective tissue cells during limb development.

## Background

Components of the limb musculoskeletal system have distinct mesoderm origins. Myogenic cells originate from somites, while components of the skeletal system originate from limb lateral plate mesoderm [1–4]. Reciprocal interactions between the different components of the musculoskeletal system are required during development to ensure a complete and functional musculoskeletal system. We designed a technique to study the molecular interactions between somitic-derived tissues (muscles) and lateral-plate-derived tissues (skeletal components and attachments) during limb development, using the chick model.

Skeletal muscle development relies on two successive and overlapping waves of myogenesis. Embryonic myogenesis establishes the scaffold of muscles, while foetal myogenesis ensures muscle growth and maturation [5, 6]. Both embryonic and foetal myogenesis rely on muscle progenitors that express the Paired homeobox transcription factors Pax3 and Pax7 [7]. In chick embryos, Pax3+/Pax7+ muscle progenitors delaminate from the ventro-lateral lips of dermomyotomes and migrate into limb buds, where they proliferate and organize in two dorsal and ventral muscle masses [8]. Muscle progenitors enter the myogenic program via the sequential activation of the bHLH myogenic regulatory factors (MRFs), Myf5, MyoD, and Myogenin. MyoD promotes cell cycle exit with the direct activation of the cyclin-dependent kinase inhibitor p57^kip2^ [9–11]. Muscle differentiation involves a cell fusion process to give rise to multinucleated muscle fibres [5]. Once the muscle differentiation process has started, muscle masses split progressively to give rise to individualised limb skeletal muscles [12].

In parallel to skeletal muscle development, the skeleton formation occurs. The skeleton is attached to muscles via tendons and connective tissues. Skeletal elements are linked together with ligaments. Limb skeletal elements (cartilage/bone) and attachments (tendons, ligaments and connective tissues) are derived from limb lateral plate [1–4, 13]. During limb development, cartilage differentiation is initiated by the condensation of the mesenchyme in the centre of the limb bud [14] surrounded by dorsal and ventral muscle masses. Consequently, in early limb buds myogenic and cartilage cells are located in different limb regions and do not physically interact. In contrast, tendon and connective tissue cells are mixed with myogenic cells in dorsal and ventral limb regions [13, 15, 16].

Despite distinct mesoderm origins, the development of skeleton, muscle and attachments is highly coordinated to ensure a proper functional musculoskeletal system, in which tendons transmit the force generated by muscles to bones in order to allow movement [13, 16]. Limb skeleton development initiates independently of muscles [17], although mechanical forces generated by muscle contraction are needed for further bone development [17, 18]. Consistent with their different mesoderm origins, limb muscles and tendons initiate developmental process independently of each other. However, tendons require functional skeletal muscles to further differentiate [13, 19–21]. Connective tissue differentiates from limb bud mesenchymal cells and will provide structural support to other limb tissues. Genetic modification of limb muscle connective tissues affects limb muscle formation and patterning [15, 16, 22].

The electroporation technique is one current method to study gene function during chick development. Since the establishment of the in ovo electroporation in 1997 [23], numerous laboratories have been using this technique to misexpress genes in chick embryos. Over the years the electroporation technique has been applied to different embryonic tissues, mostly neural tubes [24–28] and somites [29, 30], but also in aortic endothelial cells [31]. The electroporation technique has been a useful tool to study chick limb development, to target either the muscle lineage with limb somite electroporation [32–35] or limb mesenchyme with limb lateral plate electroporation [36–38]. Strategies have been developed to improve gain and loss-of function experiments in chick embryos using the electroporation technique [39–41]. However, most studies are based on electroporation with the use of transient vectors. Due to the episomal expression of these vectors, the electroporated cells failed to maintain the transgene expression more than 48 to 72 h after electroporation [42–44]. It therefore prevented any study at late stages of development. Consequently, techniques based on transposon-mediated gene transfer have been designed to obtain stable gene integration into the genome and to study late developmental stages in chick embryos. To date, three transposon systems are available, the Tol2 transposon system that originate from the medaka fish [42, 45], the PiggyBac and Sleeping Beauty systems [46, 47]. PiggyBac and Tol2 transposons have been proven to be efficient in chick cells [48].

In this report, we designed new vectors that stably and simultaneously express two fluorescent proteins, Tomato and GFP (green fluorescent protein) using the Tol2 transposons and the viral T2A system. These new vectors driving the bicistronic expression of the two fluorescent proteins under the control of a ubiquitous promoter were used to stably misexpress genes in the muscle lineage or in limb lateral plate derived-tissues, using chick tissue electroporation. We also designed new stable vectors containing muscle-specific promoters to target myogenic cells at different steps of the differentiation process. Chick limb somite electroporation with these muscle-specific vectors allowed us to stably and simultaneously co-express two proteins at different steps of myogenesis. We believe that all these new vectors combined with the electroporation technique are powerful tools to study tissue interactions during limb development in chick embryos.

## Results and discussion

### Stable and bicistronic expression of TdTomato and EGFP fluorescent reporter proteins using the Tol2 transposon and the viral 2A peptide systems

**Fig. 1:**
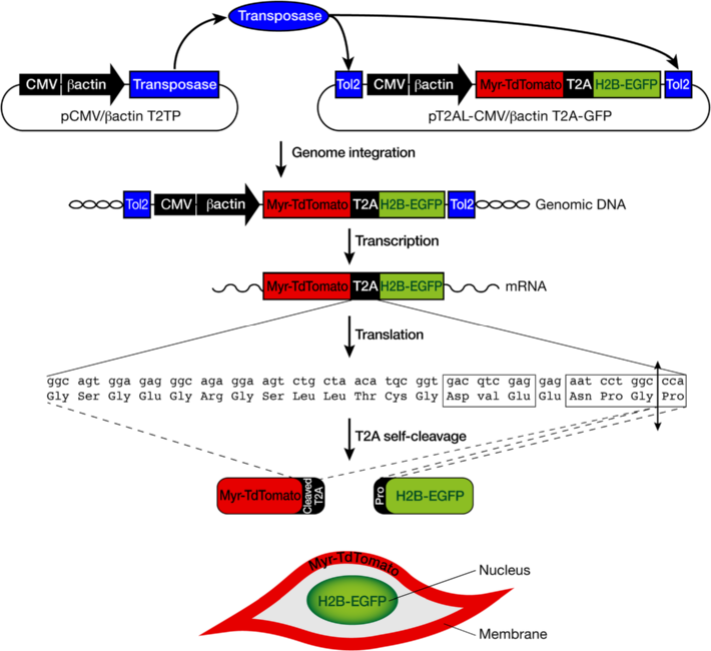
Schematic representation of the 2A peptide system in Tol2-based vectors. Schematic representation of a vector containing the transposase under the control of a ubiquitous CMV/βactin promoter and a vector containing a cassette with two reporter fluorescent genes TdTomato and EGFP separated by the T2A peptide under the control of a CMV/βactin promoter, between the minimal Tol2 transposons. The transfection into chick cells of both vectors allows the stable transposition of the transgene (CMV/βactin promoter-TdTomato-T2A-EGFP cassette) into the chick genome. The TdTomato-T2A-EGFP cassette is transcribed under the control of the CMV/βactin promoter and then translated. At the level of the translation process, the T2A peptide will be self-cleaved between the two amino acids, Gly and Pro (double arrow), following a consensus sequence (boxed). The 19 first amino acids of the cleaved T2A peptide remains fused to the C-terminus of the TdTomato, while the Pro amino acid is added to the N-terminus of the GFP. With the 2A peptide system, one single mRNA is transcribed that produces two proteins in stoichiometric proportions. TdTomato is targeted to the membrane due to a myristoylation signal and EGFP is targeted to the nucleus due to a H2B sequence. This leads to expression of both proteins in two different subcellular compartments

We previously designed stable vectors based on the Tol2 transposon, which allowed us to stably misexpress genes-of-interest in chick embryos [44]. However, with this stable vector set, we had to co-electroporate two recombinant vectors, one expressing the gene-of-interest and one expressing a fluorescent reporter protein in order to follow the ectopic gene-of-interest in ovo. One limitation was that both recombinant vectors were not systematically co-integrated into the chick genome preventing any analyses at a cellular level. The IRES (Internal Ribosome Entry Site) is a system that drives the expression of two genes using an unique vector [43, 49]. However, the IRES system has proven some failures regarding the expression levels of the second protein [50]. The viral 2A peptide system has been generated to circumvent the problem of different protein expression levels and has been shown to allow the simultaneous expression of several proteins in stoichiometric proportions [50–52]. The 2A peptides were found in viruses that used these peptides to mediate protein cleavage [51]. 2A peptides are small peptides that are self-cleaved between the last two amino acids (Gly and Pro) following a rare and conserved consensus motif (Fig. 1) [51, 52]. After translation, proteins are produced in stoichiometric proportion from one unique transcript [50–52]. There are several available 2A peptides derived from different viruses, which display high self-cleavage efficiency [50, 51]. The T2A peptide originating from the insect *Thosea asigna* virus 2A was used to generate a bicistronic cassette, which links the TdTomato (Tandem dimer Tomato) and the EGFP (enhanced Green Fluorescent Protein) proteins [52]. We cloned this bicistronic cassette under the control of a ubiquitous promoter, the CMV/βactin promoter. The CMV/βactin promoter, comprising the CMV (cytomegalovirus) enhancer and the chick β-actin promoter, has been proven to be highly efficient for transient transgenesis in chick embryos [24, 34, 35, 53]. In order to stably integrate this cassette into the chick genome, we inserted the CMV/βactin promoter and the bicistronic cassette into the stable vector based on the Tol2 transposon system [44]. Because TdTomato and EGFP are targeted to the membrane and nucleus, respectively [52], this system allows the stable integration into genomic DNA and the bicistronic expression of the two proteins with comparable expression levels in different subcellular compartments (Fig. 1).

### Chick limb somite electroporation with the PT2AL-CMV/βactin-Tomato-T2A-GFP vector

**Fig. 2:**
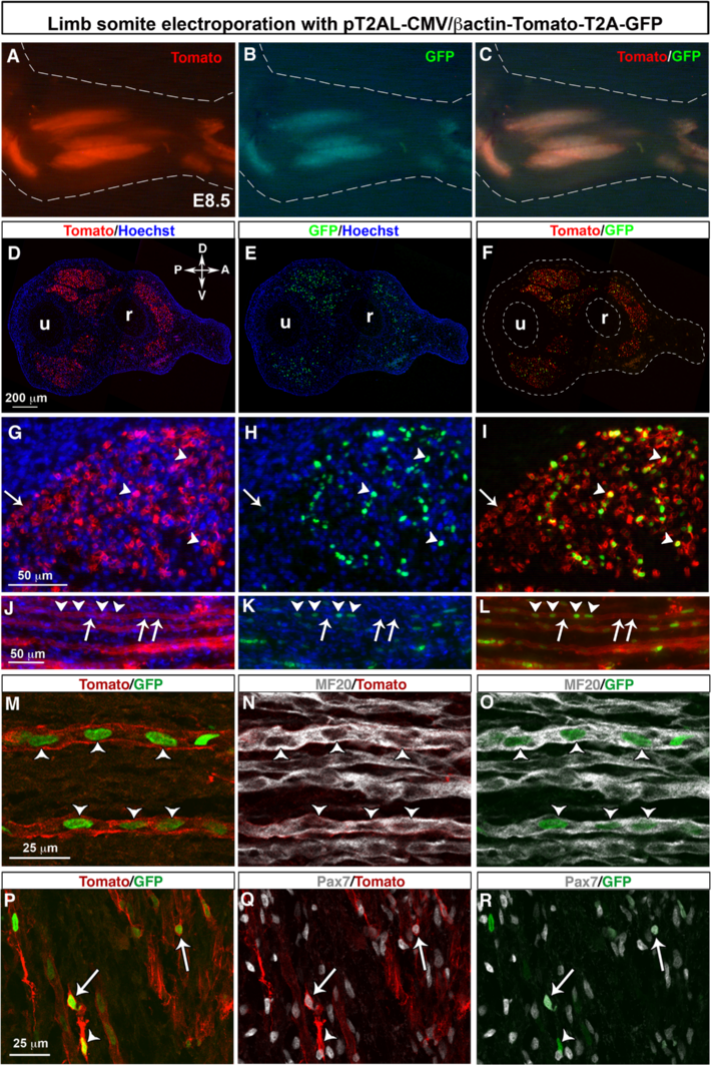
Stable and bicistronic expression of Tomato and GFP fluorescent proteins in forelimb muscles following chick limb somite electroporation. Forelimb somites of E2.5/HH15 chick embryos were electroporated with the pT2AL-CMV/βactin-Tomato-T2A-GFP stable vector containing the Tomato-T2A-GFP cassette under the control of a general promoter. Six days after electroporation, at E8.5, forelimbs were collected for wholemount visualisation (**a**-**c**), immunostaining on transverse (**d**–**i**) or longitudinal (**j**–**r**) limb sections. Both Tomato and GFP fluorescent proteins were expressed in forelimb muscles, visualised in whole mount embryos (**a**–**c**). The Tomato and GFP expression was visualised in all limb muscles on transverse limb sections (**d**–**f**). Higher magnifications of muscle transverse sections showed that GFP+ nuclei were associated with Tomato in membrane (**g**–**i**, arrowheads). However, Tomato was not always associated with GFP due to the multinucleated statute of muscle fibres and membrane fluidity (**g**–**i**, arrows). Longitudinal muscle sections showed electroporated muscle fibres displaying Tomato fluorescence in sarcolemma with only a subset of GFP+ myonuclei (**j**–**l** arrowheads). GFP- myonuclei are arrowed (**j**–**l** arrows). **m**–**r** Electroporated muscle cells co-expressing both Tomato at the membrane and GFP in nuclei were observed in MF20+ muscle fibres (**m**–**o**, arrowheads) and in Pax7+ progenitors (**p**–**r**, arrows)

DNA electroporation was performed to hypaxial lips of dermomyotomes of limb somites of E2.5/HH15 chick embryos in order to target limb myogenic cells [35] with the stable and bicistronic PT2AL-CMV/βactin-Tomato-T2A-GFP vector. Six days after limb somite electroporation with this vector set, we observed the expression of both Tomato and GFP proteins in forelimb muscles of E8.5/HH34 chick embryos (Fig. 2a–c). Forelimb transverse sections of electroporated chick embryos showed the expression of Tomato and GFP in limb muscles and no expression in the lateral plate derived-tissues such as cartilage elements (Fig. 2d–f). Consistent with the cellular compartment addressing sequences, Tomato and GFP were observed at the cellular membrane (Fig. 2g, arrowheads) and in nucleus (Fig. 2h, arrowheads), respectively, in limb muscles. All GFP+ nuclei were surrounded by Tomato + membrane (Fig. 2i, arrowheads). However, GFP- nuclei could be observed in Tomato + muscle fibres (Fig. 2g–l, arrows). We believe that only a few myoblasts were sufficient to target Tomato to the entire sarcolemma of muscle differentiated and multinucleated cells, due to membrane fluidity and the fusion process of muscle cells (Fig. 2j–l). It is likely that non electroporated myoblasts fuse to electroporated muscle cells. This explained why we observed myonuclei displaying GFP (Fig. 2j–l, arrowheads) and myonuclei displaying no GFP expression (Fig. 2j–l, arrows) in muscle fibre sarcolemma displaying red fluorescence (Fig. 2j–l). The use of the generic CMV/βactin promoter leads to Tomato and GFP expression in MF20+ cells (Fig. 2m–o, arrowheads) and in Pax7+ muscle progenitors (Fig. 2p–r, arrows), following limb somite electroporation.

We conclude that limb somite electroporation with the pT2AL-CMV/βactin-Tomato-T2A-GFP vector leads to GFP and Tomato bicistronic and stable expression in muscle progenitors and muscle differentiated cells, in chick limbs. Either fluorescent protein can be replaced by a gene-of-interest since the two proteins are produced in stoichiometric proportion. Membrane Tomato fluorescence will be adequate to follow electroporated myotubes even though not all myonuclei are targeted with GFP. Nuclear GFP will allow the targeting of electroporated nuclei in muscle cells.

### A p57 Muscle Regulatory Element combined with the βactin promoter drives bicistronic expression of Tomato and GFP in myoblasts and muscle fibres

**Fig. 3:**
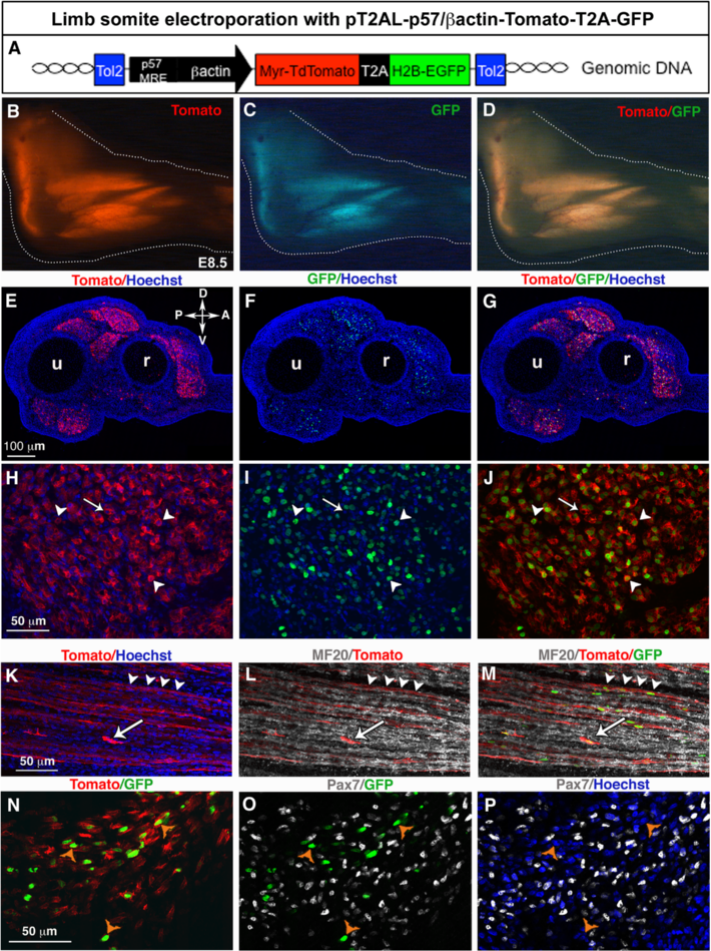
Stable and bicistronic expression of Tomato and GFP fluorescent proteins in myogenic cells following chick limb somite electroporation with a muscle-specific regulatory element. **a** Forelimb somites of E2.5/HH15 chick embryos were electroporated with the PT2AL-p57/βactin-Tomato-T2A-GFP stable vector containing the Tomato-T2A-GFP cassette under the control of the p57MRE muscle-specific regulatory element. Six days after electroporation, at E8.5, forelimbs were collected for wholemount visualisation (**b**–**d**), immunostaining on transverse (**e**–**j**, **n**–**p**) or longitudinal (**k**–**m**) limb sections. **b**–**d** Both Tomato and GFP fluorescent proteins were expressed in forelimb muscles, visualised in whole mount embryos. **e**–**g** The Tomato and GFP expression was visualised in limb muscles on transverse limb sections. Higher magnifications of muscle transverse sections showed a general co-localisation of GFP+ nuclei with membrane Tomato (**h**-**j**, arrowheads). However, Tomato was not always associated with GFP due to the multinucleated statute of muscle fibres and membrane fluidity (**h**–**j**, arrows). Tomato and GFP expression was observed in MF20+ muscle fibres (**k**–**m**, arrowheads) and in MF20- cells (**k**–**m**, arrows). **n**–**p** Nuclear GFP or membrane Tomato expression driven by the p57 regulatory element was barely observed in Pax7+ muscle progenitors (**n**–**p**, arrowheads). Scale bars, (**e**–**m**) 50 μm (**k**–**p**)

Limb somite electroporation with a generic promoter targets gene expression in muscle progenitors before their migration to the limb. In order to study gene function after the migration step, we designed a stable vector containing the Tomato-T2A-GFP cassette under the control of the p57MRE/βactin promoter (Fig. 3a). The p57^kip2^ cyclin-dependent kinase inhibitor is directly activated by Myod in myoblasts in vitro and in vivo [9–11]. The addition of this mouse regulatory sequence to a generic chick βactin promoter should drive gene expression in myoblasts. The pT2AL-p57/βactin-Tomato-T2A-GFP vector was electroporated into forelimb somites of chick embryos. Six days after electroporation, we observed that limb muscles displayed red and green fluorescence, in E8.5 chick embryos (Fig. 3b–d). This was confirmed on transverse forelimb sections where we observed that muscles were expressing both Tomato and GFP (Fig. 3e–g). GFP always co-localised with Tomato showing that the GFP nuclei were always associated with Tomato + membranes (Fig. 3h–j, arrowheads). However, as for the PT2AL-CMV/βactin-Tomato-T2A-GFP vector (Fig. 2), Tomato + muscle fibres could be observed with GFP+ and GFP− nuclei (Fig. 3h–j, arrows). Longitudinal muscle sections of E8.5 electroporated forelimbs revealed that the p57MRE-βactin promoter drove the expression of both fluorescent proteins in MF20+ muscle fibres (Fig. 3k–m, arrowheads) as well as in mononucleated MF20- cells (Fig. 3k-m, arrows), but rarely in Pax7+ muscle progenitors (Fig. 3n–p, arrowheads).

We conclude that the p57MRE-βactin promoter drives transgene expression mainly in myoblasts and muscle fibres. Combined with somite electroporation, this vector set targets gene expression in muscle cells at step downstream of muscle progenitors.

### Stable and bicistronic expression of Tomato and GFP fluorescent proteins in differentiated muscle cells using the Myosin Light Chain promoter

**Fig. 4:**
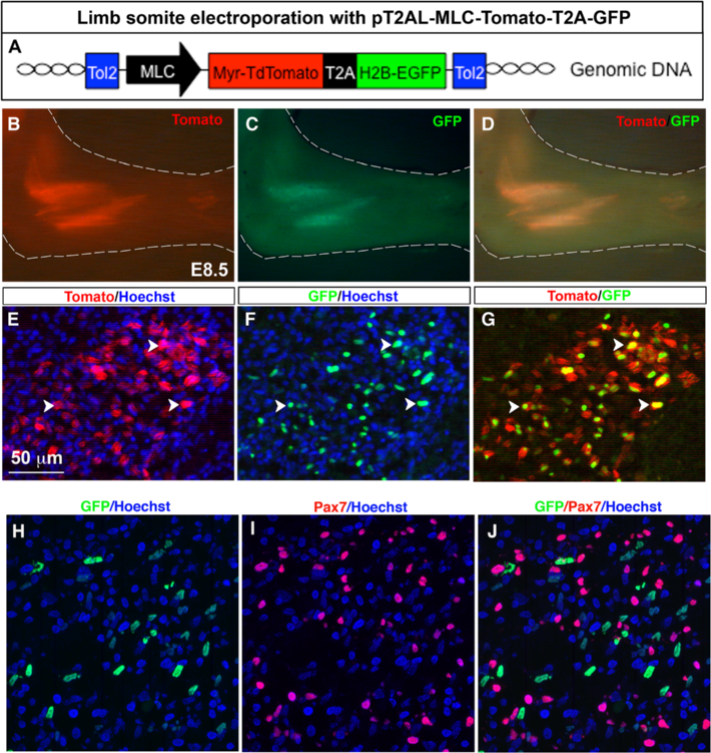
Stable and bicistronic expression of Tomato and GFP fluorescent proteins in differentiated muscle cells following chick limb somite electroporation with a myosin light chain promoter. **a** Forelimb somites of E2.5/HH15 chick embryos were electroporated with the PT2AL-MLC-Tomato-T2A-GFP stable vector containing the Tomato-T2A-GFP cassette under the control of the myosin light chain (MLC) promoter. The MLC promoter drives gene expression in differentiated muscle cells. Six days after electroporation, at E8.5, forelimbs were collected for wholemount visualisation (**b**–**d**) or immunostaining on transverse limb sections (**e**–**j**). Most of the GFP+ nuclei were surrounded by a Tomato + sarcolemma (**e**–**g**, arrowheads). **h**–**j** GFP+ nuclei were never observed in Pax7+ cells. Scale bars = 50 μm

**Fig. 5:**
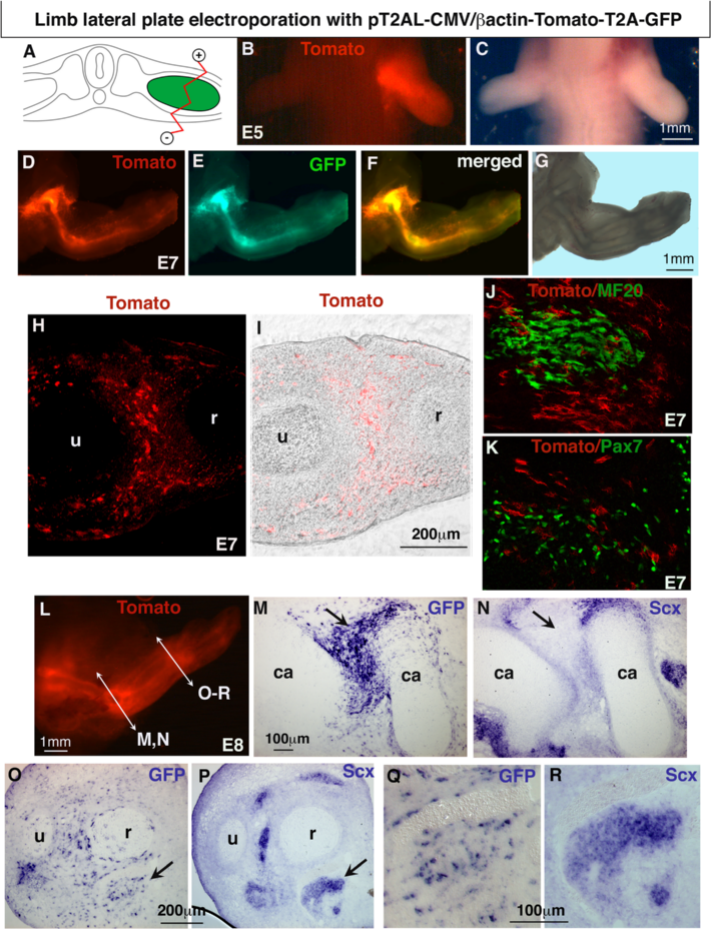
Stable and bicistronic expression of Tomato and GFP fluorescent proteins in chick forelimbs following limb lateral plate electroporation. **a** Limb lateral plate of E2/HH13 chick embryos was electroporated with the pT2AL-CMV/βactin-Tomato-T2A-GFP stable vector containing the Tomato-T2A-GFP cassette under the control of a general promoter. Forelimbs were collected for wholemount visualisation of Tomato or GFP, 3 (**b**, **c**), 5 (**d**–**g**) and 6 (**l**) days after electroporation. **h**, **i** Transverse sections of forelimbs 5 days after electroporation to visualise Tomato expression in limbs. **j**, **k** Tomato fluorescence was observed in limb connective tissues and never observed in muscle differentiated (**j**) or progenitor (**k**) cells. **l** Forelimbs were collected for wholemount visualisation of Tomato 6 days after electroporation. **m**–**r** In situ hybridisation experiments to transverse limb sections of the electroporated forelimb shown in L, with GFP (**m**, **o**, **q**) and Scx (**n**, **p**, **r**) probes at the level of the proximal (**m**, **n**) and distal (**o**–**r**) forearm. **m**, **n** and **o**, **p** are adjacent sections. **m**, **n** shows strong GFP expression in regions surrounding cartilage elements (arrow). **o**–**r** GFP expression in tendons. **q**, **r** is a high magnification of a tendon shown in (**o**, **p** arrowed). ca, cartilage, u, ulna, r, radius

There is evidence that differentiated muscle cells signal to muscle progenitors to regulate muscle growth during development [11, 54, 55]. One option to study the molecular dialogue between muscle fibres and muscle progenitors is to specifically misexpress genes in differentiated muscle cells. Consequently we established another stable vector, in which the Tomato-T2A-GFP cassette was inserted under the control of the mouse Myosin Light Chain (MLC) promoter (Fig. 4a). The mouse MLC promoter drives transgene expression in differentiated muscle cells [44]. In E8.5 electroporated chick embryos, we observed red and green fluorescence in limb muscles, indicating that both Tomato and GFP proteins were expressed (Fig. 5b-d). Both Tomato and GFP proteins were observed in limb muscles on transverse limb sections (Fig. 5e-g). GFP+ nuclei were associated with Tomato + labelling (Fig. 5e–g, arrowheads). As for the CMV/βactin and the p57/βactin promoters, we observed Tomato + muscle fibres with GFP+ and GFP- nuclei (Fig. 5e–g, arrows), due to the spread of Tomato in sarcolemma of multinucleated muscle cells. As expected and previously shown for the MLC promoter [44], Tomato and GFP fluorescence was never in Pax7+ muscle progenitors (Fig. 5h–j).

We conclude that the stable and muscle-specific vector pT2AL-MLC-Tomato-T2A-GFP leads to bicistronic expression of Tomato and GFP proteins in differentiated muscle cells in chick limb. Replacing either fluorescent protein encoding genes with a gene-of-interest will efficiently drive transgene misexpression in muscle differentiated cells.

### Chick limb lateral plate electroporation with the generic CMV/βactin promoter drives bicistronic expression of Tomato and GFP proteins in cartilage, tendon and connective tissues

In order to target the non-myogenic cells of the limb musculoskeletal system, we electroporated the pT2AL-CMV/βactin-Tomato-T2A-GFP in the forelimb lateral plate of E2/HH13 chick embryos (Fig. 5a). Three days after electroporation, fluorescence was observed throughout the forelimb (Fig. 5b, c). Five days after electroporation both Tomato and GFP proteins were diffusely expressed in chick limbs (Fig. 5d–g). Notably, a high fluorescence was observed in cartilage elements (Fig. 5d–f). Transverse limb sections showed a general Tomato expression in forelimbs (Fig. 5h, i), expression which did not delineate muscles in contrast to somite electroporation with the same vector (Fig. 2). In limb muscles, Tomato was never observed in MF20+ differentiated muscle cells (Fig. 5j) nor in Pax7+ muscle progenitors (Fig. 5k). We believe that cells displaying Tomato fluorescence in limb muscles following lateral plate electroporation correspond to muscle connective tissue cells. GFP transcripts could be observed in cartilage regions (Fig. 5l–n, arrows). GFP transcripts could also been observed in tendons, which are labelled with the key tendon marker Scleraxis (Scx) (Fig. 5o–r, arrows).

We conclude that limb lateral plate electroporation with the pT2AL-CMV/βactin-Tomato-T2A-GFP vector leads to biscistronic and stable transgene expression in lateral plate-derived tissues, such as cartilage, tendon and muscle connective tissues.

## Conclusion

**Fig. 6:**
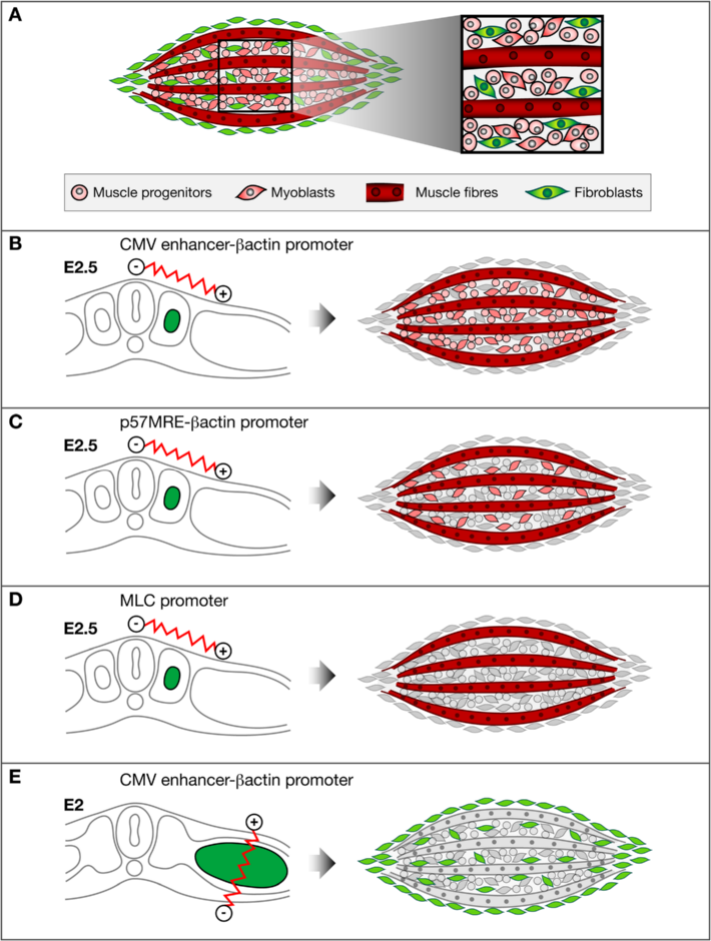
Schematic representation of myogenic and non-myogenic cells in muscles targeted with limb somite or lateral plate electroporation with stable vectors producing reporter genes under the control of different promoters. **a** Representation of the somitic- (red) and lateral-plate-derived (green) lineages in limb skeletal muscles. Skeletal muscles are composed of myogenic cells at different steps of differentiation somitic-derived (red) and of muscle connective tissue cells lateral plate derived (green). At a specific time point, muscle progenitors, mononucleated muscle cells or myoblasts and differentiated multinucleated muscle cells coexist in muscles. Myogenic cells originate from limb somites (**b**–**d**), while connective tissue cells derive from limb lateral plate (**e**). Limb somite electroporation with stable vectors containing different promoters. **b** Limb somite electroporation with a stable vector containing a generic CMV/β-actin promoter drives reporter gene expression in myogenic cells at all steps of muscle differentiation. **c** Limb somite electroporation with a stable vector containing a p57 regulatory element and the chick β-actin promoter drives reporter gene expression in myoblasts and differentiated muscle cells. **d** Limb somite electroporation with a stable vector containing a MLC promoter drives reporter gene expression in differentiated muscle cells. **e** Lateral plate electroporation with a stable vector containing a CMV/β-actin promoter drives reporter gene expression in muscle connective tissue cells (inside muscle and surrounding muscle) and tendons

In summary, we designed new vectors that stably and simultaneously express two distinct proteins. Limb muscles are composed of myogenic cells originating from somites and of connective tissue cells derived from lateral plate (Fig. 6a). Myogenic cells in muscles are at different steps of the muscle differentiation process, ranging from muscle progenitors, myoblasts to muscle fibres (Fig. 6a). Limb somite electroporation (Fig. 6b–e) with a generic (Fig. 6b) or muscle-specific promoters (Fig. 6c, d) will target all myogenic cells (Fig. 6b), myoblasts and muscle differentiated cells (Fig. 6c) or only muscle differentiated cells (Fig. 6d), respectively. Lateral plate electroporation with a generic promoter (Fig. 6e) target muscle connective tissue cells, while somite lateral plate electroporation with a generic promoter target myogenic cells (Fig. 6b). This provides us with tools to study the molecular interactions between cellular components of muscles. We believe that these new vectors combined with tissue-specific electroporation techniques are powerful tools to study chick limb development.

## Methods

### Chick embryos

Fertilized chick eggs from a commercial source (JA57 strain, Dangers, France) were incubated at 38.5 °C. Embryos were staged according to days in ovo. For early stages, the following day numbers and HH (Hamburger and Hamilton) stages [56] are equivalent: E2/HH13, E2.5/HH15 and correspond to 20 and 25 somite stages, respectively.

### Establishment of recombinant vectors

The pT2AL-MLC-Tomato-T2A-GFP plasmid was obtained as following: The Myr-TdTomato-T2A sequence was amplified by PCR from the plasmid pCS2-TdTomato-2A-GFP [52]. To facilitate subsequent cloning, one XhoI site was added to the forward primer and one BstBI site was added to the reverse primer. The purified PCR product was then inserted into pCRII-TOPO (Invitrogen) and a clone with Tomato downstream of SP6 promoter was selected, giving rise to a plasmid named TOPO/Tomato. H2B-GFP was amplified by PCR from the plasmid pCS2-TdTomato-2A-GFP [52]. A BstbI site was added to the forward primer and one PmlI site and one ClaI site were added to the reverse primer. The purified PCR product was then inserted into pCRII-TOPO (Invitrogen) and a clone with GFP downstream of SP6 promoter was selected, resulting in a plasmid called TOPO/GFP. Next, both TOPO/Tomato and TOPO/GFP were digested with BstbI and NotI. The T2A sequence was then inserted into TOPO/Tomato using the T4 DNA ligase (New England Biolabs) to generate a plasmid named TOPO/Tomato-T2A-GFP. The Tomato-T2A-GFP cassette was then excised from TOPO Tomato-T2A-GFP using EcoRV and XhoI and cloned into the pT2AL200R150G [57] previously digested with ClaI (blunt-ended using Fermentas T4 DNA polymerase) and XhoI. The resulting plasmid was named pT2AL-Tomato-T2A-GFP. The Myosin Light Chain (MLC) mouse promoter was removed from the pT2K-MLC-Fgf4 plasmid (previously described in [44]) using NcoI and XhoI. Both extremities were then blunt-ended using T4 DNA polymerase (Fermentas). The MLC promoter was next blunt ligated to TOPO GFP previously digested with XbaI made blunt. A clone with the MLC promoter inserted with ApaI in 5’ and XhoI in 3’ was selected resulting in a plasmid called TOPO/GFP/MLC. Both TOPO/GFP/MLC and pT2AL-Tomato-T2A-GFP were digested with ApaI and XhoI. MLC was inserted into pT2AL-Tomato-T2A-GFP to obtain pT2AL-MLC-Tomato-T2A-GFP.

The pT2AL-CMV/βactin-Tomato-T2A-GFP plasmid was obtained as followed: The pT2AL-MLC-Tomato-T2A-GFP plasmid was digested with ApaI (blunt-ended using Fermentas T4 DNA polymerase) and SphI to remove the MLC promoter. The MLC promoter was then replaced by the CMV-βactin promoter (the chick βactin promoter downstream of a CMV enhancer), which was excised from the CMV-βactin-EGFP [35] using SalI (blunt-ended) and SphI to generate the pT2AL-CMV-Tomato-T2A-GFP plasmid.

The pT2AL-p57/βactin -Tomato-T2A-GFP plasmid was obtained as followed: The p57MRE regulatory sequence was excised from pSK-p57MRE plasmid [11] by digestion with Spe1 and SacII. The CMV enhancer of the pT2AL-CMV/βactin-Tomato-T2A-GFP plasmid was excised by Acc1 and SnaBI and replaced with the p57MRE using blunt ligation with the Rapid DNA Ligation Kit (Roche). The generated plasmid was named the pT2AL-p57/βactin-Tomato-T2A-GFP.

### Electroporation

Limb somite electroporation was performed as previously described [35]. The DNA solution was systematically composed of the Tol2 stable vectors and the transient transposase vector CMV/βactin-T2TP, which allows the stable integration into the chick genome. The concentration of the different vectors was between 1.5 and 2 μg/μL and of 1/3 for the CMV/βactin-T2TP. DNA was prepared in solution containing carboxymethyl cellulose 0,17 %, fast green 1 %, MgCl_2_ 1 mM and PBS 1X in water.

Lateral plate electroporation was performed as followed: Stage HH13–15 (E2) chick embryos were windowed following standard techniques in preparation for electroporation [58]. PBS without Ca^2+^/Mg^2+^ was applied to the embryo. A capillary was backfilled with DNA solution, which was injected under 200 Pa pressure (injection duration 0.1–0.5 s and compensatory pressure 15–25 Pa) (Femtojet, Eppendorf) into the embryonic coelom, to fill completely the anterior to posterior extent of the forelimb territory. The negative electrode (0.8 mm diameter tungsten rod with a 4-mm length and 2-mm exposed surface) was inserted into the yolk and positioned beneath the forelimb field, approximately 2 mm below the embryo. A 0.8 mm diameter platinum rod with a 1-mm exposed tip served as the positive electrode and was positioned above the forelimb field with an approximate distance of 3 mm. A wave pulse train consisting of 50 V, five pulses, 20 ms duration with a 200 ms interpulse interval was delivered via TSS20 electroporator and EP21 current amplifier (Intracel). Embryos were returned to 37.5 °C for the remaining incubation period. DNA solution was composed of pT2AL-CMV/βactin-Tomato-T2A-GFP (1-3 μg/μL) and CMV/βactin-T2TP at a molar ratio of 1:5–1:10, diluted in a mix containing PBS without Ca^2+^/Mg^2+^ and Fast Green 0.005 %. This ratio resulted in persistent gene expression in the embryonic limbs during foetal development.

### Immunohistochemistry

Experimental forelimbs were fixed in paraformaldehyde 4 % overnight at 4 °C and processed for cryostat sections (12 μm). Immunohistochemistry was performed as previously described [59]. The monoclonal antibodies MF20 that recognizes sarcomeric myosin heavy chains and Pax7 that recognizes muscle progenitors, developed by D.A. Fischman and A. Kawakami, respectively, were obtained from the Developmental Studies Hybridoma Bank developed under the auspices of the NICHD and maintained by The University of Iowa, Department of Biology Iowa City, IA 52242. After overnight incubation with the primary antibody at 4 °C, biotinylated secondary antibodies (Anti-Mouse IgG2b from Southern Biotech; Anti-Mouse IgG1 from Jackson ImmunoResearch laboratories) were applied for 1 h at room temperature, followed by a 45 min incubation with Cy5-Streptavidin (Invitrogen). Hoechst (Molecular Probes) staining was performed with a dilution of 1/20000 in PBS 1X for 10 min at room temperature.

### In situ hybridization

In situ hybridization experiments were performed for *GFP* and *Scx* probes, as previously described [35].

### Image capturing

Images of the wholemount electroporated limbs were acquired with a Leica stereo-macroscope equipped with a Leica DFC300 camera. After immunohistochemistry, sectioned samples images were captured using a Nikon epifluorescence microscope, a Leica DMI600B inverted microscope or a Leica SP5 confocal system.

## Abbreviations

CMV: cytomegalovirus
EGFP: enhanced Green Fluorescent Protein
HH: Hamburger and Hamilton
IRES: Internal Ribosome Entry Site
MLC: Myosin Light Chain
MRFs: Myogenic Regulatory Factors
T2A peptide: *Thosea asigna* 2A peptide
TdTomato: tandem dimer Tomato
